# Substrates, Plants, and Their Combinations for Water Purification of Urban Household Aquaponics Systems

**DOI:** 10.3390/ijerph191610276

**Published:** 2022-08-18

**Authors:** Yi-Han Wu, Qing-Feng Chen, Jia-Nan Wang, Ting Liu, Wei-Yi Zhao

**Affiliations:** 1 College of Geography and Environment, Shandong Normal University, Jinan 250358, China; 2 Shandong Analysis and Test Center, Qilu University of Technology (Shandong Academy of Sciences), Jinan 250014, China

**Keywords:** aquaponics, urban households, substrate materials, aquatic plants, water purification, microbes

## Abstract

To make full use of urban household balcony space, an urban aquaponics system for balconies was constructed to investigate the purification effects of four different substrates (volcanic stone, ceramic pellets, ceramic rings, and nanorods) and six plants (mung bean sprouts, hollow cabbage, water celery, lettuce, leek, and water chestnut) on fish culture wastewater. Through the determination of contaminants such as nitrogen and phosphorus and through the use of 16SrDNA sequencing technology, the substrate material and plant combinations with the best purification effects were screened. The results show that volcanic stone and nanorods have strong purification capacities. Compared to the other substrate types, there were more unique bacterial species on the surface of volcanic stone, among which amoeba species were the most dominant (92.42%). Among the six tested plant species, mung bean sprouts had the highest contribution to nitrogen uptake (94.96%), and water chestnut had the highest contribution to phosphorus uptake at 12.07%. Finally, the combination of nanorods and water celery was the best at purifying the wastewater. This study provides a theoretical basis and new ideas for the construction of urban aquaponics systems on balconies, which will help to achieve green farming and the efficient utilization of water resources.

## 1. Introduction

In traditional home aquaculture, the ammonia nitrogen produced by the accumulation of fish metabolites is toxic to fish, and the water in the tank must be regularly changed to maintain normal fish growth [1]. This aquacultural wastewater is usually discharged directly as domestic sewage, which not only causes certain environmental pollution, but also wastes a substantial amount of water resources. As a new type of compound cultivation technology, aquaponics, reduces water pollution by organically combining fish farming and vegetable cultivation, and it also achieves the goal of green farming. Thus, aquaponics is an environmentally friendly farming system that also saves resources [2].

With the increasing standard of living, urban residents are pursuing healthier and greener organic food, and it has become popular to have aquacultural farms on balconies at home in China. In particular, aquaponics systems in which the wastewater from fish farming is used to grow vegetables, are promising for at-home aquaculture. In aquaponics systems, pollutants such as ammonia, nitrogen, and phosphate are transformed into nutrients that are non-toxic to fish and can promote vegetable growth after decomposition by microorganisms attached to the substrate material and vegetable roots. Through this process, the wastewater from fish tanks on balconies can be recycled efficiently, and urban residents can access healthy organic vegetables at any time.

Most aquaponics culture models utilize greenhouse aquaculture, and there are few systems available for urban households. Lennard and Leonard compared the purification effects of three different aquaponics subsystems in hydroponic trials and concluded that [3] in terms of biomass and yield, gravel bed technology was superior compared to float and nutrient film technology, respectively. Domestic aquaponics systems mainly utilize industrial models and are used to a certain extent in Chinese cities, including Beijing, Guangdong, and Shandong.

Currently, there are three main aquaponics methods applied in urban households: direct flotation, nitrification filtration, and separated drip irrigation. The ones used in the study were separated drip irrigation. (1) The direct floating method is simple and easy to operate. Generally, put hydroponic vegetable seedlings are directly put in foam boards and other floating bodies for hydroponics, but the disadvantage of this method is that the roots of vegetables are easily damaged by fish. (2) The nitrification filtration method was proposed by Graber and Junge [4]. The breeding water body and the planting system are connected to the nitrification filter bed, the waste water is filtered by the nitrification filter bed, the vegetables are cultivated on the nitrification bed, and the filtered water is recycled through the hydroponic vegetable system as a nutrient solution to supply vegetable nutrients. The water absorbed nutrients by vegetables returns to the breeding pool, forming a cycle. (3) The separation drip irrigation method makes it relatively simple to distribute the water from the breeding system to the planting area by drip irrigation, after the substrate filtration and the plant absorption. However, this cultivation mode has strict requirements on the cultivation substrate and requires the size of peas. In addition, some foreign scientists have also carried out related research on microorganisms. For example, Kasozi et al. studied the effect of *Bacillus* on lettuce growth and root-associated bacteria [5], and they elucidated that the addition of *Bacillus* to hydroponic systems could effectively alleviate nutrient deficiencies in water bodies, improve water quality, and improve the bacterial community composition.

However, most studies have only investigated the purification effect and growth conditions of plants in aquaponics systems; the purification effect and microbial communities of the aquaponics substrate material have been less studied. In addition, very few domestic and foreign studies are applied to urban families, and there are gaps for the screening of substrate and vegetable combinations. Therefore, this study compares the purification effects of four substrates, selected the substrate material and plant combinations that are most suitable for different water quality conditions, and reveals the microbial community structure on different substrate surfaces in order to provide a theoretical basis and novel ideas for constructing urban aquaponics systems on balconies.

## 2. Materials and Methods

This section explains the materials and methods used in the system.

### 2.1. Experimental Materials

Four different substrates were used in this experiment, namely, a volcanic stone (vesuvianite), nanorods, ceramic pellets (haycite), and ceramic rings. The substrates were placed at a height of 10 cm in filter tanks separated according to particle size. Then, 40 ornamental fish, such as koi and goldfish, were cultured in the fish tank, and fish were feed using Shandong Italian brand three-in-one fish feed; the main ingredients are fish meal and shrimp powder. The feeding time was 9 a.m. every day; the feeding amount was about 10% of the weight of the fish [6].

### 2.2. Experimental Method and Design

The composition of the system and the water quality test method are divided into three main aspects below.

#### 2.2.1. Experimental Design

**Figure 1:**
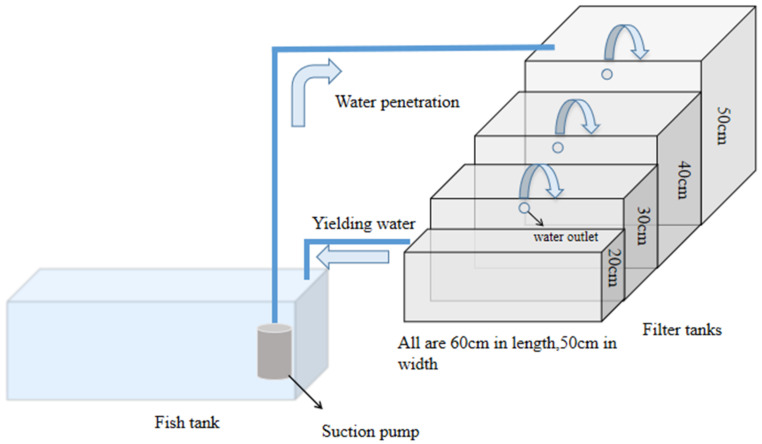
Diagram of the tested aquaponics system.

As shown in Figure 1, the test setup consisted of three main parts: a fish tank of length × width × height = 120 cm × 45 cm × 45 cm; a four-stage filtration tank of 60 cm length, 50 cm width, and heights of 50, 40, 30, and 20 cm; and a PVC pipe connection between the fish tank and the filtration tank. The test setup was placed on an urban sealed balcony with a natural light source. The filter tanks were placed at a greater height than the fish tank so that the water in each filter tank could directly flow back to the fish tank by gravity. The water in the fish tank and each filter tank was maintained at a height of 5 cm from the top edge of the container. In this recirculation system, the effluent from the previous filter tank was used as the initial water for the next filter tank. To ensure a constant water level, any evaporated water was replaced in a seven-day cycle.

The experiment was conducted in two parts. (1) The first part was to place only fish fry and substrate in the system to determine the purification effects of different substrates on wastewater. Water quality was tested in a seven-day cycle (with the seventh day recorded as the initial measurement day; see figures), and water from the outlet of each filter tank was collected as a water sample. As the system was a closed circuit circulation system, the concentration change law of the water quality index in each filter water sample of each tank is based on its own comparison—that is, the concentration changes in the four filters in the test cycle. At the end of the measurement period, microbiological measurements were performed on the surfaces of the substrates. Volcanic stone, nanorod, ceramic pellet, and ceramic ring substrates were labeled as C1, C2, C3, and C4, respectively. (2) The second part of the experiment was to place different plants in the substrate-lined filter tanks to investigate the purification efficiency of different plants and the combined purification effect of substrates and plants on wastewater.

First, water samples without fry were tested as initial data. Then, fish fry was placed in the tank and water quality monitoring was continued. Water samples were measured for ammonia nitrogen, phosphate, total phosphorus, chemical oxygen demand (COD), and other water quality indicators. The removal effect of each substrate on wastewater pollutants was expressed as:

(1) $$removal\;rate\;\left(\%\right)=\frac{T_{i}-T_{k}}{T_{i}}\times 100\%$$

where *T_i_* is the mass concentration of pollutants in the water before purification and *T_k_* is the mass concentration of pollutants in the water after substrate purification.

In this experiment, the substrate was measured from 12 March to 23 April 2021. Plants were introduced into the system on 23 April. Six plant types were grown: mung bean sprouts, water celery, hollow cabbage, lettuce, leek, and water chestnut (Ling). Plants were secured on a floating board and placed in a filter. Water chestnut was planted in volcanic stone filter, water celery was planted in the nanorod filter, water spinach was planted in the ceramic filter, and mung bean sprouts were planted in the ceramic ring filter. The growth cycles of water celery and mung bean sprouts are short, so were planted after leeks and lettuce in the corresponding filters. After harvesting, the root and stem lengths of each plant were measured. The harvested plants were dried at 106 °C for 30 min and 60 °C for 120 min, ground, and their contributions to nitrogen and phosphorus uptake in wastewater were calculated. The utilization rate was found as follows:

(2) $$utilization\;efficiency\;\left(\%\right)=\frac{T_{i}}{S_{i}-S_{k}}\times 100\%$$

where *T_i_* indicates accumulation of nitrogen and phosphorus in the plants (mg), *S_i_* indicates the initial content of nitrogen or phosphorus in the water (mg), and *S_k_* indicates the final content of nitrogen or phosphorus in the water (mg).

#### 2.2.2. Experimental Methods

The water quality indices of the water samples were measured based on [7]. Ammonia nitrogen content and COD_cr_ were measured based on [8]. Total phosphorus and total nitrogen were measured based on [7]. Total nitrogen content in the plants and total phosphorus content were measured based on [9]. At the end of the experiment, equivalent quality matrix material was removed from the bottom of the four filters; and into 500 mL of ultra-pure water for 24 h, the shock samples were placed using the microbial filter. The microbial enrichment on the filter membrane was immediately taken into the laboratory for cryopreservation, and then sent to gene a sequencing company (Beijing Biotechnology Co., Ltd., Beijing, China) for 16s rDNA sequencing. The 16s rDNA sequencing was performed to determine which microbial species were present on the substrate surface.

#### 2.2.3. Data Analysis

The data were analyzed and plotted with Microsoft Excel 2015 and Origin Pro. The correlations between the accumulation of nitrogen and phosphorus in plants and the contributions of nitrogen and phosphorus uptake were analyzed. The correlations between the water quality indexes were analyzed. Microbial diversity analysis was performed using mothur software [10].

## 3. Results and Discussion

### 3.1. Purification Effects of Different Substrate Materials on Wastewater

The removal capacities of nitrogen and phosphorus from wastewater were analyzed with the matrix.

#### 3.1.1. Effects of Different Substrates on the Removal of NH_4_^+^-N

**Figure 2:**
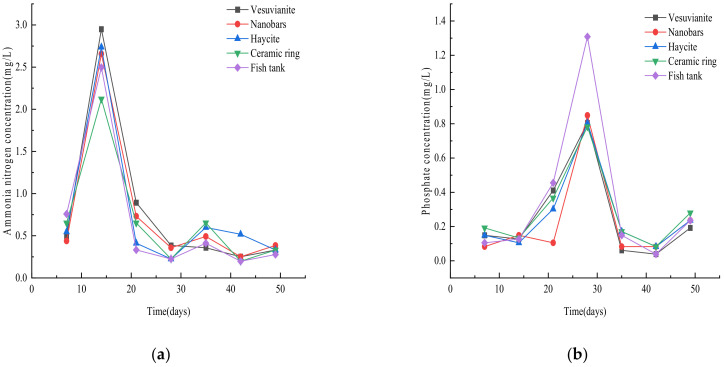
Variation in (**a**) ammonia nitrogen and (**b**) phosphate mass concentration in each filter tank and the fish tank.

Due to the death of some fish fry, there is excess bait, and the excreta is not all consumed by fish. The concentration of ammonia nitrogen in fish farming water thereby increases over time [11]. When the NH_4_^+^-N content in the water is too high (>5 mg/L), fish will die; thus, ammonia nitrogen is considered a major “killer” in aquaculture. The effect of the substrate material on the removal of ammonia nitrogen is therefore an important criterion [12]. Figure 2a shows that the mass concentration of ammonia nitrogen in ever substrate, and the fish tank suddenly experienced an increase around the 14th day of the experiment, with a maximum concentration of 2.95 mg/L in the volcanic rock substrate. This phenomenon occurred because the experiment had just started, so the system was not yet stabilized and the content of nitrifying bacteria was low. Additionally, the water in the fish tank was first discharged into the volcanic rock filter, which was located at the greatest height; these factors led to the accumulation of ammonia nitrogen in the system. Analysis of the changes in the mass concentration of ammonia nitrogen during the first 42 days showed that the volcanic rock, nanorods, and ceramic pellets were the most effective at purifying ammonia nitrogen. After a brief increase in the mass concentration of ammonia nitrogen on day 14, the mass concentration of ammonia nitrogen in the volcanic rock filter steadily decreased. On day 35, the ammonia nitrogen concentration in all the filters slightly increased, except for the volcanic stone filter. Based on an overall analysis of Figure 2a, the starting concentrations of ammonia nitrogen were similar in all filters, suggesting that the matrix plays an important role in maintaining the cleanliness of the water. Of the substrate types, the volcanic stone had the most stable removal effect on ammonia nitrogen. Ammonia nitrogen is the most common pollutant in water bodies, so it can be seen that volcanic stone has a significant effect on purifying sewage.

#### 3.1.2. Effects of Different Substrates on Phosphate Removal

In addition to nitrogen, phosphorus is often an excess nutrient in aquatic systems [12]. By day 21, the phosphate mass concentration in the nanorod substrate filter tank had decreased to 0.105 mg/L, but the phosphate contents in the other substrate filter tanks and the fish tank were increasing. This occurred because the filter tanks with ceramic pellets or ceramic rings contained water that had flowed from the nanorod filter tank, so the phosphate mass concentration in the water of the ceramic-containing filter tanks increased following the filtration effect of the nanorod substrate. Additionally, the mass concentrations of phosphate in these filters were lower than those in the fish tank and volcanic stone substrate filter tank. On the 28th day, the phosphate content in the water of the four filter tanks and the fish tank began to decrease. Figure 2b shows that the mass concentration of phosphate in the fish tank wastewater gradually decreased after filtration through the four substrates, and the four substrates had a clear purification effect on phosphate.

#### 3.1.3. Effects of Different Substrates on the Removal of Total Phosphorus

From Table 1, we can see that the mass concentration of total phosphorus in each water sample fluctuated greatly. As the test proceeded, the mass concentration of total phosphorus in each water sample increased. In the first 14 days, the mass concentrations of total phosphorus in the fish tank and the volcanic stone filter tank increased most obviously, but after another week, the total phosphorus mass concentration in the volcanic stone filter had decreased by 83.4%. The average purification efficiency of the volcanic stone substrate for total phosphorus reached 83.3%, which was much higher than that of any other tested substrate material. Therefore, volcanic stone had the best purification effect for total phosphorus.

#### 3.1.4. Variation in the COD_cr_ of Different Substrate Filter Cells

**Figure 3:**
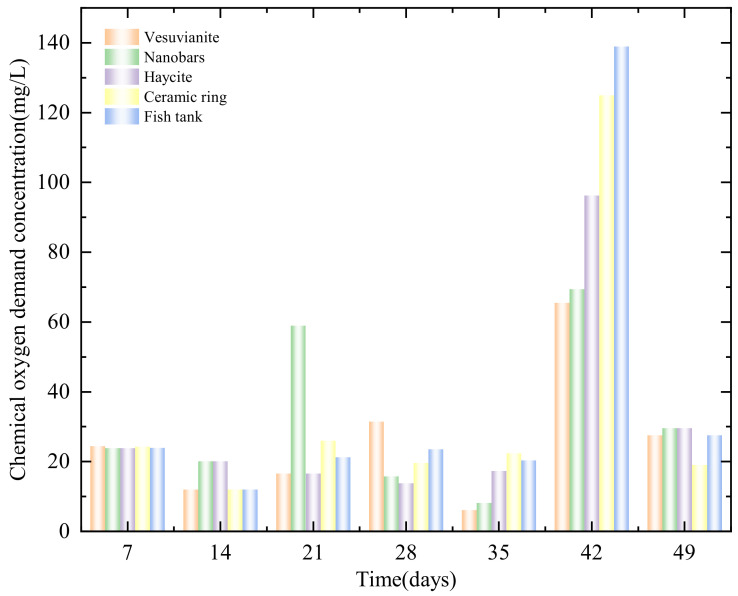
Changes in the chemical oxygen demand in each filter tank and the fish tank.

Organic matter in the water body is decomposed into inorganic matter by the microorganisms attached to the substrate surface, which reduces the consumption of dissolved oxygen in the water body and promotes nitrification [13]. In this experiment, each filter substrate had a rough surface, so all substrates were conducive to microbial growth. On the 35th day, the COD_cr_ concentration in each tank dropped to a very low level, and the nitrogen and phosphorus concentrations in the water were also low (Figure 3). On the 42nd day, the COD_cr_ in the fish tank and each filter tank increased sharply due to the accumulated effects of fish metabolism and fish food. At this point, the COD_cr_ concentration in the fish tank was the highest among the five tanks. However, after one week, the COD_cr_ rapidly decreased by 80.2%, 84.8%, 69.2%, 58.0%, and 57.4% in the fish tank, ceramic ring, ceramic pellet, volcanic stone, and nanorod substrate filter tanks, respectively. As shown in Table 1, the volcanic stone substrate filter had the least change in COD_cr_ and the highest COD_cr_ removal rate, which shows that volcanic stone can play an important role in maintaining the cleanliness of aquacultural tanks.

#### 3.1.5. Purification Capacity of Different Substrate Materials

**Figure 4:**
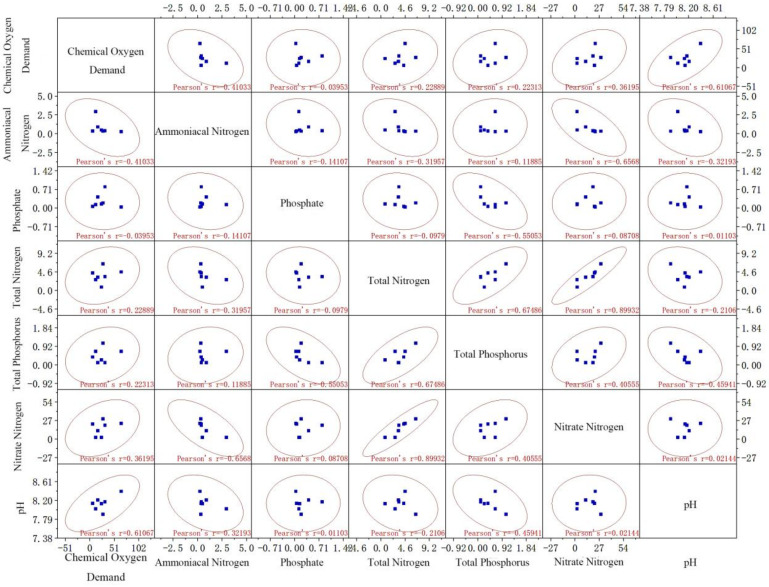
Correlation analysis of water quality indices using volcanic stone as the substrate.

Table 1 shows that the ammonia nitrogen purification capacities of different substrates were in this order: volcanic stone > ceramic ring > ceramic pellets > nanorods. For phosphate purification, capacities were ranked as: volcanic stone > ceramic rings > nanorods > ceramic pellets. For COD, the capacities were ranked as: volcanic stone > ceramic pellets > ceramic rings > nanorods. For total phosphorus, the capacities were ranked as: volcanic stone > ceramic rings > nanorods > ceramic pellets. Therefore, compared with the other three substrates, volcanic stone had the best purification effects on ammonia nitrogen, phosphate, total phosphorus, and COD: purification efficiencies reached 88.8%, 95.1%, 83.3%, and 63.1%, respectively. Furthermore, the purification effect of volcanic stone was the most stable. However, nanorods had the best purification effect for total nitrogen. Considering the comprehensive purification effects of volcanic stone, this substrate was further used in a correlation analysis of the water quality indices (Figure 4).

A matrix surface for nitrification bacteria and other microbial growth provides sufficient growth space. Li PX study also proved that the high adsorption of nitrogen by zeolite and ceramite may be attributed to their large combined specific surface area [14]; therefore, it is good substrate, and this aquatic vegetable collocation combination will get twice the result with half the effort. There are a large number of substrate materials used to purify wastewater on the market, mainly including zeolite, volcanic stone, ceramsite, brick slag, sponge iron, grain husk, activated carbon [15,16,17], etc., which all have good adsorption capacities.

The purification with a fish symbiosis system has a matrix, aquatic vegetables, microorganisms, extracellular enzymes, root secretions, and other factors. This experiment mainly studied the water purification abilities of different matrixes and aquatic vegetables; and microorganisms, extracellular enzymes, and root secretion to the system to remove nitrogen and phosphorus still need to be explored in further experiments. Further study on the extracellular enzymes, root secretions, and microbial community; and the wastewater purification mechanism and response relationship in the system also need study. The results can help people to better choose substrates to form a family fish and vegetable symbiosis system.

#### 3.1.6. Correlation Analysis of Water Quality Indices with Volcanic Stone Substrate

Ammonia nitrogen was negatively correlated with nitrate-nitrogen (Figure 4), indicating that the ammonia nitrogen in the water was converted to nitrate-nitrogen by nitrifying bacteria and microorganisms. Therefore, the volcanic stone substrate played a purifying role in the aquacultural system. This is also one of the reasons why the volcanic stone matrix filter is effective for ammonia nitrogen removal. The correlations of other indicators were low, which shows that other factors in the system may affect the water quality purification of the volcanic stone matrix, which can be further explored in future studies.

### 3.2. Effectiveness of Different Plants on Pollutant Removal

#### 3.2.1. Effects of Different Plants on Ammonia Nitrogen Removal

**Figure 5:**
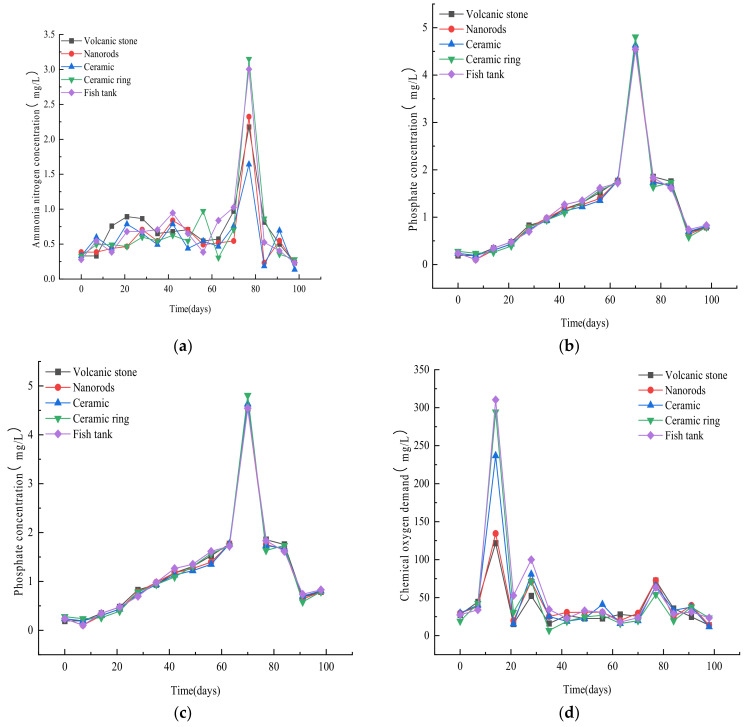
Changes in ammonia nitrogen (**a**), phosphate (**b**), nitrate-nitrogen (**c**), and chemical oxygen demand (**d**) concentrations in each filter tank and the fish tank after plants were added to the aquaponics system. Day 0 was 23 April 2021.

The plant growth dates were as follows: water chestnut, days 7 to 98 in the volcanic stone filter; water celery, days 0 to 35 in the nanorod filter; days 56 to 98 for leeks in the nanorod filter; water spinach, days 0 to 35 in the ceramic filter; mung bean sprouts, days 0 to 7 in the ceramic ring filter; and days 35 to 70 for lettuce in the ceramic ring filter. As shown in Figure 5a, the ammonia nitrogen concentration showed small fluctuations in the first 70 days (starting time: 23 April 2021) across the different tanks. Due to the short growth period of mung bean sprouts, the removal of both nitrogen and phosphorus from the wastewater was not obvious. However, water celery planted in the tank with the nanorod filter resulted in good absorption of ammonia nitrogen. Although the ammonia nitrogen concentration in the filter was still increasing, the growth rate was slow and the ammonia nitrogen concentration was more stable. Water celery planted in the filter with ceramic pellets was slightly less effective at purifying the wastewater. In this tank, the ammonia nitrogen concentration showed two small fluctuations during the first 35 days after planting, with the highest increase being 0.348 mg/L. After the water celery planted in the nanorod filter was harvested, leeks were planted. During the leek growth period, a large increase in ammonia nitrogen occurred on day 77, but the increase in the ammonia nitrogen concentration in the nanorod filter tank was less than those in the ceramic ring filter tank and the fish tank. Therefore, the combination of nanorods with water celery and leeks was the most effective at purifying the water of ammonia nitrogen.

#### 3.2.2. Effects of Different Plants on Phosphate Removal

From Figure 5b, it can be seen that there was little difference in the phosphate concentrations among the filter tanks. There was an increasing trend at the beginning of the experimental period; and there was a particularly sharp increase in phosphate concentration on the 70th day. On this day, the highest phosphate concentration was observed in the nanorod filter tank (4.805 mg/L). It is presumed that due to the harvesting of lettuce, some dead branches and residual leaves fell into the filter tank, resulting in elevation of nitrogen and phosphorus in water. When the phosphate concentration in the system sharply increased, the content of ammonia nitrogen also sharply increased. Furthermore, the phosphate content in the other filter tanks was similar to that in the nanorod filter tank. Therefore, there was no considerable difference in the removal of phosphate by the planted vegetables.

#### 3.2.3. Variations in COD_cr_ in Filter Tanks with Different Plants

Due to the absorption and utilization of pollutants in the wastewater by the plants, the COD_cr_ was maintained at a stable low level of about 50 mg/L for most of the experimental period. Furthermore, the demand for nutrients such as nitrogen and phosphorus in the wastewater was low because each plant was still in the seedling stage at the beginning of the experiment [18]. However, the COD_cr_ increased sharply on the 14th day in each filter tank, and the highest concentration (310.4 mg/L) was observed in the fish tank (Figure 5d). It is presumed that the pollutants in the four-stage filter system accumulated gradually, resulting in a sudden increase in COD_cr_ in the system.

#### 3.2.4. Variations in Nitrate-Nitrogen Concentration in Filter Tanks with Different Plants

In aquaponics systems, the nitrate-nitrogen converted to ammonia by nitrifying bacteria can be absorbed and used by plants as nutrients [19]. In this experiment, the nitrate-nitrogen content in the aquaponics system steadily increased, starting at the early stage of the experiment (Figure 5c), likely because the uptake of nitrate-nitrogen by plants was lower than the production of nitrate-nitrogen. However, the nitrate-nitrogen concentration in each filter tank considerably dropped between day 28 and day 91, which corresponded to the period of rapid growth for water celery and hollow cabbage. As the rapid growth period of these plants was about two weeks before their harvest, there was a continuous decrease in nitrate-nitrogen, coupled with plant adsorption of nitrate in water bodies [20]. The synergistic filtration effect of the four plants (Water celery, water cabbage, water chestnut, mung bean sprouts) increased the efficiency of nitrate-nitrogen utilization in the water column, and during days 29–91, the nitrate-nitrogen contents decreased by 71.9%, 99.1%, 71.2%, and 71.2% for the volcanic rocks, nanorods, ceramic rings, and nanorod filter tanks, respectively. Although the nitrate-nitrogen concentration in all four filter tanks considerably decreased, the nitrate-nitrogen concentration in the nanorod filter tank exhibited the greatest decrease: up to 99.1%. The water celery planted in the nanorod filter tank was in a better state of growth compared to that grown in the other filter tanks, allowing the water celery to fully absorb and utilize the nitrate-nitrogen in the tank.

#### 3.2.5. Purification Capacities of Different Plant Species for Nitrogen and Phosphorus

The uptake capacities and contribution rates of nitrogen and phosphorus varied greatly among plant species, and there were also large differences in the uptake capacities of nitrogen and phosphorus by different parts of the same plant species (Table 2). The contribution rates of nitrogen uptake for the six plants ranged from 55.39% to 94.96%, and the contribution rates of phosphorus uptake ranged from 0.54% to 12.07%. Compared with other studies [21], the reason for the relatively high contributions of nitrogen and phosphorus in this study may have been that the experiment was conducted in spring, when the plants grow better and have a higher demand for nitrogen and phosphorus [22], resulting in relatively high contributions of the plant roots to nitrogen and phosphorus in the water column.

In this experiment, the contributions of nitrogen and phosphorus uptake by different parts of the same plant varied greatly. The contribution of root nitrogen uptake ranged from 4.75% to 52.48%, and root phosphorus uptake ranged from 0.02% to 0.81%. Similarly, the contribution of stem and leaf nitrogen uptake ranged from 31.87% to 65.61%, and that of stem and leaf phosphorus uptake ranged from 0.29% to 12.07%. This was observed because the removal of nitrogen in the water column occurs not only through nitrification and denitrification by microorganisms, but also through plant uptake. Aquatic plants can transport oxygen to the roots to provide a suitable environment for nitrification and denitrification, and phosphorus is removed from the water column through both plant uptake and substrate and root adsorption [23].

The correlations between the accumulation of nitrogen and phosphorus in plants and the contributions to nitrogen and phosphorus uptake were analyzed. There was a strong correlation between the accumulation of phosphorus and the contribution of plants to phosphorus uptake (Pearson’s R = 0.885; *p* < 0.01), whereas there was no significant correlation between the accumulation of nitrogen and the contribution of plants to nitrogen uptake. These results demonstrate that the accumulation of phosphorus can reflect the ability of plants to purify the water, and also indicate that the purification of phosphorus by plants mainly occurs through absorption. In contrast, the purification of nitrogen in the water occurs through nitrification and denitrification by water microorganisms in addition to direct root uptake [24]. In summary, based on the water purification ability of the substrate materials and the plant growth state, it can be concluded that the combination of nanorods and water celery is the best tank design.

### 3.3. Analysis of Microbial Diversity on Different Substrate Surfaces

The microbial core flora and the diversity of the substrate surface can reflect the microecological situation of an aquaponics system [25]. Table 3 shows the abundances and diversity of microorganisms in different samples. The Shannon and Simpson indices reflect the species richness, and the larger the Shannon index and the smaller the Simpson index, the higher the species diversity in the samples. The Chao1 and ACE indexes usually indicate the abundance of species [10], and in Table 3, both the Chao1 and ACE index rank C1 > C4 > C2 > C3, indicating that the species abundance was the highest on the surface of the volcanic substrate (C1). These results show that the microbial diversity was highest on the surface of the nanorod substrate, whereas the abundance of microbial species was highest on the surface of the volcanic rock substrate.

**Figure 6:**
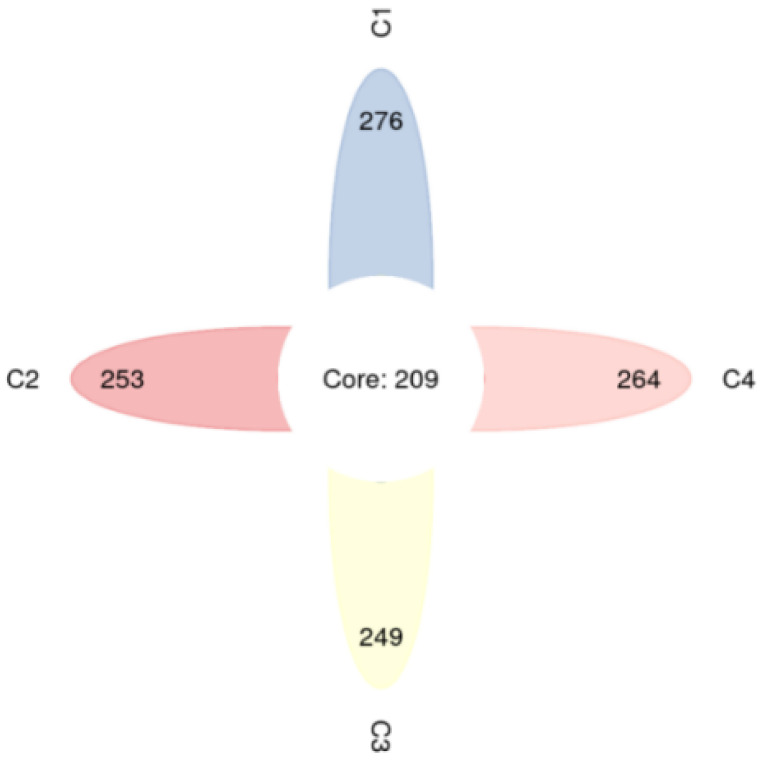
Venn diagram of the operational taxonomic units for microorganisms growing on the four substrate types. C1, volcanic stone; C2, nanorods; C3, ceramic pellets; C4, ceramic rings.

From the Venn diagram of operational taxonomic units (OTUs) presented in Figure 6, it can be seen that volcanic stone substrate (C1) had more microbial species attached to its surface, and the number of unique species on the volcanic stone substrate surface was higher than those of the other three substrates [26]. This was presumably due to the large number of surface pits, large specific surface area, and stable physicochemical properties of volcanic stone that allow it to be a suitable living environment for microorganisms.

**Figure 7:**
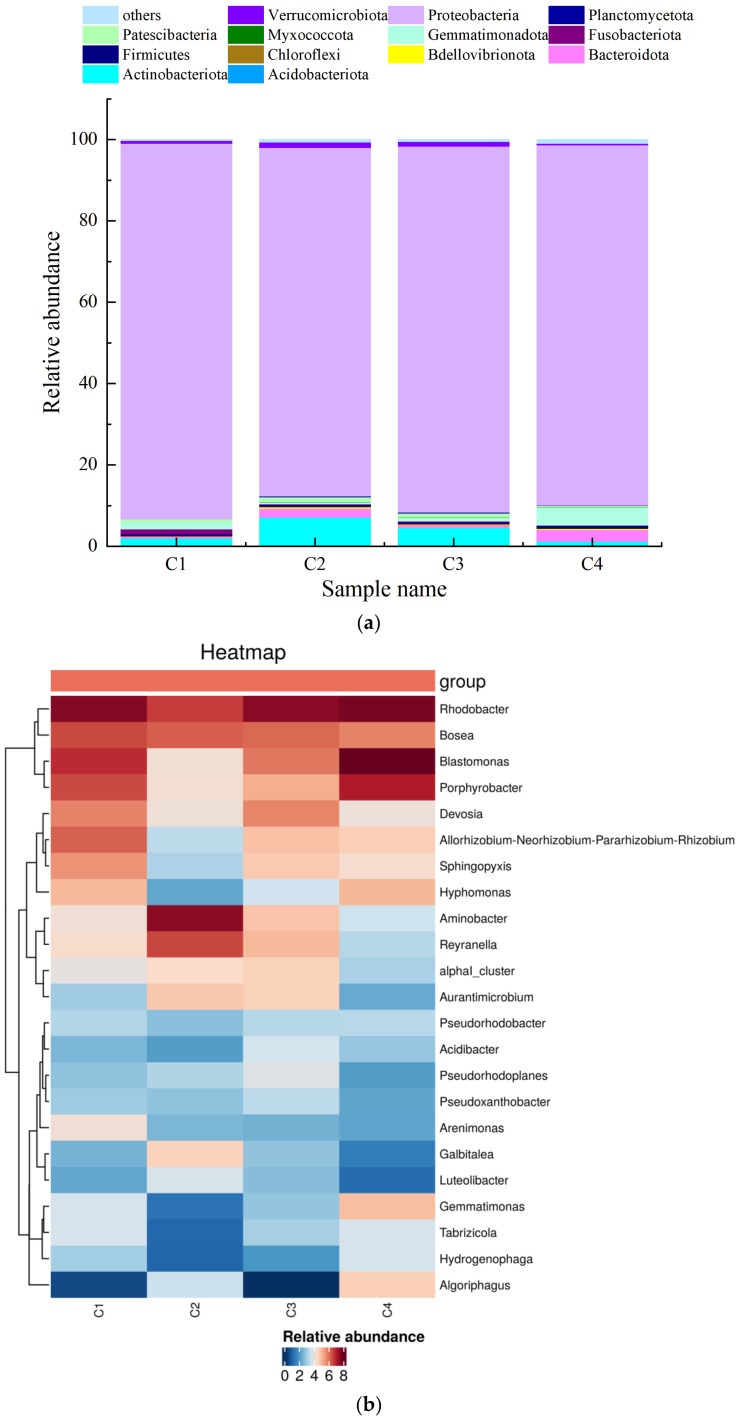
Histogram of relative abundance of species at the phylum level (**a**) and heat map of clusters at the genus level (**b**) for microorganisms living on the surface of different substrates (C1, volcanic stone; C2, nanorods; C3, ceramic pellets; C4, ceramic rings).

The differences in the abundance of microorganisms among the substrates at the genus level can be seen in Figure 7a. There was high abundance of *Rhodobacter* spp. in C1, C3, and C4, and a more even distribution and stronger correlation between genera in C1. There was higher abundance of microorganisms in comparison to the results of previous studies. Figure 7b shows the classification of the microorganisms in the samples at the phylum level, and it can be observed that Proteobacteria were the most dominant bacteria on all four substrates: C1 (92.42%) > C3 (90.03%) > C4 (88.64%) > C2 (85.72%). In C1, the most dominant bacteria were Proteobacteria and Gemmatimonadota. The most dominant bacteria in C2 were Proteobacteria, Actinobacteriota, and Bacteroidota. The most dominant bacteria in C3 were Proteobacteria and Actinobacteria. The most dominant bacteria in C4 were Proteobacteria, Actinobacteriota, and Gemmatimonadota.

In summary, volcanic rock substrates are more likely to have more microorganisms attached due to their rough surfaces [27]. The microorganisms on the surface of the volcanic rock substrate were characterized by high species richness and low diversity. It can be seen in Figure 6 and Figure 7 that the microorganisms on the volcanic rock substrate surface were numerous and mostly endemic, and these endemic OTUs may be related to the better purification effect of the volcanic rock substrate. However, the microbial diversity on the surface of the volcanic stone substrate was low. It is likely that since the volcanic stone substrate was the first filter through which the fish tank water passed, some impurities in the fish tank inhibited the growth of certain microorganisms. Similarly, Zhao et al. showed that the organic pollutants in a water body can have a destructive effect on the microbial community [27]. The genus *Rhodobater*, which dominated in C1, C3, and C4, is a class of photosynthetic bacteria that can efficiently purify the water and promote denitrification [28]. Ammonia nitrogen can be oxidized to nitrate-nitrogen by nitrifying bacteria, nitrate-nitrogen can be reduced to molecular nitrogen through denitrification, and the molecular nitrogen can then enter the atmosphere. In particular, red bacteria can effectively reduce the concentration of ammonia nitrogen in the water through denitrification. Furthermore, they can use a variety of organic compounds as carbon sources and electron donors for photoheterotrophic growth under anaerobic conditions, they play an important role in photosynthesis and carbon cycling in aquaponics systems, and they are involved in nitrogen and phosphorus removal from wastewater. Therefore, the concentrations of ammonia nitrogen in the volcanic stone, ceramic pellet, and ceramic ring filter tanks changed significantly due to the presence of these bacteria. In addition, Proteobacteria and Bacteroidetes were also abundant on each substrate. The abundance of Proteobacteria was highest in C1, and this was closely related to the low concentrations of nitrogen, phosphorus, and organic matter in this filter type, as these factors play important roles in the degradation of organic matter and water quality purification; therefore, it is assumed that the synergistic effect of the microorganisms promoted nitrification and thus improved the efficiency of ammonia removal [29].

## 4. Conclusions

In summary, when combining purification by substrates and plants, the combination of water celery and nanorods had the best effect on water purification and the best resulting plant growth. This study can provide a theoretical basis and suggestions for the construction of urban household aquaponic systems. In future research, the benefits of the matrix and vegetable combination or the diseases of vegetables and fish can be further explored. In addition, we should explore the influence of plant root exudates on sewage purification, which will provide the basis for the better operation of the fish and vegetables symbiosis systems.

## Figures and Tables

**Table 1:** Average purification efficiencies of different substrate materials (mean ± standard deviation).

	Volcanic Stone (Vesuvianite)	Nanorods	Ceramic Granules (Haycite)	Ceramic Rings
NH_4_^+^	88.8% ± 0.22	66.7% ± 0.19	68.2% ± 0.24	79.6% ± 0.10
PO_4_^3-^	95.1% ± 0.17	59.9% ± 0.30	59.6% ± 0.30	60.1% ± 0.29
COD	63.1% ± 0.21	53.0% ± 0.29	55.8% ± 0.29	53.3% ± 0.25
TN	−4.8% ± 0.02	27.1% ± 0.34	12.0% ± 0.20	10.9% ± 0.18
TP	83.3% ± 0.98	55.6% ± 0.53	53.6% ± 0.18	63.5% ± 0.08

**Table 2:** Contributions of six plant species to nitrogen and phosphorus uptake.

Species	Root Contribution (%)		Stem and Leaf Contribution (%)		Total Contribution of *N* Removal	Total Contribution of *p* Removal
	Contribution of Root *N* Uptake	Contribution of Root *p* Uptake	Contribution of *N* Uptake of Stem and Leaves	Contribution of *p* Uptake in Stem and Leaves		
Bean sprouts	52.48% ± 0.02	0.81% ± 0.08	42.48% ± 0.11	0.56% ± 0.91	94.96% ± 0.59	1.37% ± 0.20
water celery	23.31% ± 0.13	0.68% ± 0.34	32.07% ± 0.28	0.62% ± 0.52	55.39% ± 0.56	1.30% ± 0.12
Hollow cabbage	42.79% ± 0.44	0.69% ± 0.25	39.65% ± 0.43	0.58% ± 0.44	56.99% ± 0.49	1.17% ± 0.17
Lettuce	4.75% ± 0.28	0.02% ± 0.89	52.24% ± 0.56	1.15% ± 0.77	83.44% ± 0.34	1.27% ± 0.29
Chives	28.22% ± 0.15	0.26% ± 0.17	31.87% ± 0.76	0.29% ± 0.22	60.09% ± 0.62	0.54% ± 0.34
Water chestnut	-	-	65.61% ± 0.88	12.07% ± 0.48	65.61% ± 0.31	12.07% ± 0.48

**Table 3:** Alpha diversity analysis based on the diversity indices of microbial operational taxonomic units.

Sample Name	Colony Abundance		Bacterial Diversity	
	Chao1 Index	ACE Index	Shannon Index	Simpson Index
C1	292.5500	287.5490	3.564768	0.063534
C2	258.6500	259.3762	3.603999	0.066217
C3	253.1053	254.1199	3.574246	0.068435
C4	278.1053	273.2113	3.223093	0.099475

## Data Availability

All data generated or analyzed during this study are included in this published article.
